# Palliative care for patients with a substance use disorder and multiple problems: a study protocol

**DOI:** 10.1186/s12904-018-0351-z

**Published:** 2018-08-03

**Authors:** Anne Ebenau, Boukje Dijkstra, Marianne Stal-Klapwijk, Chantal ter Huurne, Ans Blom, Kris Vissers, Marieke Groot

**Affiliations:** 1 Salvation Army, Central Netherlands, Zandvoortweg 211; 3741 BE, Baarn, the Netherlands; 2 Nijmegen Institute for Scientist-Practitioners in Addiction (NISPA), P.O. Box 6909, 6503 GK Nijmegen, the Netherlands; 3 Tactus Addiction care, Lokatie Ripperdastraat, Ripperdastraat 8, 7511 JR Enschede, The Netherlands; 4 Network Palliative Care region Arnhem and de Liemers, Hospice Rozenheuvel, Rosendaalselaan 20, 6891 DD Rozendaal, the Netherlands; 5 Department of Anesthesiology, Radboud University Medical Center, Pain and Palliative Care, Expertise Center for Palliative Care, Internal Post 549, P.O. Box 9101, 6500 HB Nijmegen, the Netherlands

**Keywords:** Qualitative research, Palliative care, Addiction, Substance use disorder, Vulnerability, Study protocol

## Abstract

**Background:**

The specific palliative care needs and problems of patients with a substance use disorder and multiple problems, and those of their proxies, are under recognized Besides, the organization of palliative care, including the division of health care professionals’ responsibilities, is often unclear. Perspectives of patients and proxies are hardly known. We describe the outline of a study designed to explore how palliative care for patients with a substance use disorder is organized in the Netherlands and to explore problems and needs, as well as possible improvements from the healthcare professionals’, patients’ and proxies’ perspective. The aim of this protocol paper is to provide insights in ways to conduct research with vulnerable research participants and to offer a detailed description of the study design. The broader study aims to gain insight in and formulate recommendations on how to improve palliative care for patients with a substance use disorder.

**Methods:**

A qualitative study with patients, proxies and healthcare professionals. Semi**-**structured interviews will be held with 10**–**15 patients who suffer from a severe substance use disorder. They are in a palliative care trajectory and either diagnosed with a chronic or life-threatening disease or, as a result of addictive behavior, a physical deterioration without the prospect of cure. Semi**-**structured interviews will also be held with 5**–**10 proxies. Healthcare professionals, volunteers and/or ‘experts**-**by**-**experience’ (*n* = 24**–**40) will be participating in semi**-**structured group interviews. All (group) interviews will be thematically analyzed. Additionally, a strengths, weaknesses, opportunities and threats (SWOT) analysis will be applied to the group interview data with the aim to summarize and concretize the findings.

**Discussion:**

Everyone has a right to an optimal end-of-life phase of life and a dignified dying process. This study will provide valuable knowledge about palliative care for patients with a substance use disorder and explicitly bring to light the needs and problems of the patients and their proxies and healthcare professionals in a palliative care phase.

**Electronic supplementary material:**

The online version of this article (10.1186/s12904-018-0351-z) contains supplementary material, which is available to authorized users.

## Background

Although most people with a substance use disorder (SUD) recover, others will be dependent during their entire life, and thus are likely to die while still using substances [1, 2]. People with a SUD are likely to develop chronic or life-threatening conditions and to die prematurely [3–10]. SUD is often chronic, albeit intermittent, and can have far**-**reaching consequences on several life domains and often results in problems like homelessness, financial issues, loneliness or vulnerable social networks. Furthermore, it is often accompanied by other psychiatric disorders [11–19]. SUD, thus, comes with multiple problems on patients’ physical, psychosocial and existential life domains.

For the reasons above, it is surprising that the literature about palliative care for patients with a SUD and multiple problems (from now on to be referred to as SUD+) is scarce. Palliative care has been defined as “an approach that improves the quality of life of patients and their families facing the problem associated with life-threatening illness, through the prevention and relief of suffering by means of early identification and impeccable assessment and treatment of pain and other problems, physical, psychosocial and spiritual” [20]. The scarce literature tends to focus on medical and clinical issues, such as, pain assessment and management), interaction between addictive substances and medication, the extent of alcoholism in the end-of-life phase and the addictive value of some medicines used in palliative care (mainly opioids) [21–38].

Other literature, though, recognizes broad trends and identifies various reasons that may jeopardize good palliative care for patients with a SUD, such as stigmatization; lack of training and education on this subject; ineffectiveness of and tolerance for (pain) medication due to active substance use; risks of abuse of medication by patients and; patients’ distrust, non-compliance, symptom presentation and non-disclosure of use [17, 39–46]. The current literature, however, often consists of non-replicable literature reviews or is more a reflection from the opinion of authors and individual experts and based on case studies than based on rigorous and valid research results. It fails to grasp the actual care**-**experiences of patients, proxies and healthcare professionals (HCPs). It often neglects the multidimensional nature of palliative care and is from American or Canadian origin; the findings cannot always be extrapolated to West**-**Europe.

In view of the lack of evidence and expertise on palliative care for patients with a SUD, it may be worth investigating the literature of an adjacent group, the homeless. The two groups share a high premature mortality risk, severe and persistent mental illness, chronic medical issues and, social and financial issues [16, 47–54]. The literature about palliative care for the homeless shows similar barriers as the ones described above, but also shows that such care is threatened by e.g. limited resources, little support from proxies, no appropriate care site and coordination of such care. Possible consequences are inappropriate pain treatment and symptom management as well as delayed recognition or anticipation of a patient’s deterioration or a palliative care phase [55–57].

In brief: there is a great gap in our knowledge about the specific needs and experiences of patients with a SUD+ and their proxies in a palliative care phase. Furthermore, the perspectives and needs of HCPs are also little known. The research question addressed in this study is therefore: “How is palliative care for patients with a substance use disorder and multiple problems organized in the Netherlands, and what problems, needs, improvements and good examples do healthcare professionals, patients and proxies experience or suggest?”. Several subsidiary questions need to be answered:

- How is palliative care for patients with a SUD+ organized in the Netherlands, both formally and in daily practice?
- What problems and needs in palliative care and personal lives do patients with SUD+ and their proxies’ experience?
- What problems and needs do healthcare professionals experience in providing palliative care for patients with SUD+ and their proxies?
- What are the positive experiences and best practices in palliative care for patients with SUD+, their proxies and health care professionals?

The objective of this study is to contribute to more cohesive and univocal care practice and policies for patients with SUD+ and their proxies and to formulate recommendations for improvements. Additionally, the study outcomes will serve as input for an education program for Dutch HCPs. With this particular study protocol paper, we aim to provide a detailed description of the study design and the methodological approach. By doing so, we assure transparency and reproducibility. Also, we give insight in ways to conduct research within a vulnerable research population.

We provide the readers with a case study (Additional file 1) to familiarize them with the uniqueness of palliative care for patients with SUD+ with regard to, for example, high pain medication and patients’ strong autonomy and limited communication about end-of-life issues.

## Methods/Design

### Study design

This exploratory study will use qualitative research methodologies. Individual, semi**-**structured interviews will be held with patients and their proxies. HCPs, volunteers and ‘experts**-**by**-**experience’ will be participating in focus group interviews. There will be no prior relationship between the researchers and the participants. Topics of the interview guides are based on the research questions, input and expertise of the project group (consisting of people with coordination, research and clinical care experience in addiction as well as in palliative care settings), and the literature. Data collection will take 5 months maximally. Interviews will be audio-recorded and transcribed verbatim. To attain a complete overview of practice and experiences, we strive to include participants from both metropolitan areas and more rural areas. We will adhere to the COREQ checklist (COnsolidated criteria for REporting Qualitative research) for the scientific publications [58].

### Inclusion and exclusion criteria

#### Patients and proxies

We aim to interview ten to fifteen patients with a SUD. We believe that this number is enough for the exploratory goal of this study. However, if data saturation (reached when no new data, themes and coding are emerging from the interviews) will not be reached after this number of interviews, more participants will be recruited [59]. Patients will be identified and recruited by central HCPs: people with key positions within the field of palliative care for patients with a SUD+. They mostly work in the fields of SUD and/or psychiatry, but need to have some generic knowledge about palliative care. They can be clinicians or nurses and we expect that these HCPs encounter patients with a SUD+ in a palliative care phase. Central HCPs will be project group members or their colleagues or will be recruited by the main researcher (AE).

Central HCPs will be asked to screen their patients on the inclusion criteria and the information from an instruction brochure. If a patient is eligible, the central HCPs will give him or her an information brochure and read this together. The central HCP is instructed to explicitly ask if the information has been understood. If the patient agrees to participate, the central HCP will inform the researcher, who will, in turn, contact the patient by telephone to arrange inclusion. Inclusion is possible if the patient:

1. is either officially diagnosed with the DSM-V classification severe ‘substance use disorder’ or, if such a diagnosis is lacking (e.g. caused by care-avoidance), informally assessed as such by a central HCP experienced or educated in SUD, using the criteria of the DSM-V (provided in the instruction brochure) [11]. A patient could either be still an active user, recently quit or in remission, of alcohol, cannabis, cocaine, opioids (including heroin), sedatives (often benzodiazepines) and/or Gamma-hydroxybutyric acid (GHB);
2. has a serious non-reversible, life-threatening disease (either as a consequence of a SUD or not, or is suffering from progressive, severe physical deterioration as a result of active addictive behavior which will lead to death. Diagnosing a specific life-threatening disease, such as cancer, is not always the case;
3. is 18 years or older;
4. is sufficiently fluent in the Dutch language for the purpose of an interview;
5. is cognitively capable enough to answer questions;
6. understands what participation in the study involves: spending time and energy for the interview and talking about a, potentially, emotionally demanding subject.

Furthermore, the central HCP has to:

1. answer negatively to the ‘Surprise Question’:“would it surprise you if this patient would die within the next year?” Wishing to avoid selection bias through including only patients in a terminal care phase, we will explain to the central HCPs that this question does not provide an estimate of life expectancy, but serves to recognize and recruit patients with long**-**term conditions such as cancer. They are not necessarily expected to die within 12 months, but this would not be a surprise either. They, however, might benefit from palliative care services [60, 61].
2. state that he or she explicitly told the patient that cure is no option and that a palliative phase has been reached. It is obligatory that this information should not have been told very recently. Also, the patient needs to understand this information.

We believe that since central HCPs probably are close to their patients and know them well and since patients are conscious about being in a palliative care phase, patient drop-out or non-compliance for the interviews will be low.

Exclusion criteria are the following. First, non**-**physical addiction only, such as gambling, porn/sex or gaming, will not be included, because these addictions do not directly result in physical problems. Second, severe cognitive impairment. Third, dependency on opioids for medical reasons only.

We aim to include five to ten proxies; a limited number because people with a SUD tend to have few social relations [17]. The researcher will ask participating patients to suggest a proxy, someone who is closest, albeit after having checked with the central HCP whether in individual cases there might be tensions or other sensitivities which cannot be overcome. If a patient has suggested a proxy, the researcher will ask the patient if the proxy knows that the patient is in a palliative care phase. If so, the researcher will send the proxy an information brochure and contact him/her after a week for willingness to participate.

#### Professional healthcare professionals, ‘experts-by-experience’ and volunteers (HCPs+)

We aim to organize four focus groups, each consisting of six to ten professional HCPs, ‘experts**-**by**-**experience’ and volunteers (from now on to be referred to as HCPs+). Experts-by-experience are people with lived experience of SUD, however, they are in recovery. They use their former experiences, e.g. in educating HCPs or supporting patients. We will be including different professions from different specializations and work sites to cover a wide range of experiences. As the matrix shows (Table 1), we do not aim to include people from specialized units in palliative care or specialized addiction care only (Clusters A, B and C). General care **-** often provided earlier during a care trajectory **-** is equally important (Cluster D). With regard to setting or site, our strategy thus is purposive sampling: a specific selection of research units, based on particular characteristics [62]. We intend to compose one focus group per cluster. Different recruiting strategies will be used:

1. direct sampling: approaching familiar potential participants directly from the project group members’ networks;
2. respondent**-**driven sampling (a snowballing method): asking potential participants whether they know other potential participants [63] and;
3. broad to narrow sampling: asking registries and (educational) institutions or organizations to provide contact details of eligible HCPs+ or to pay attention to the study on a website or in newsletters or magazines. Also, we will post a call for participation to LinkedIn.

**Table 1:** Sites of ‘experts-by-experience’

	Specialized palliative care settings	Settings with a generic palliative care approach (not-specialized)
Specialized SUD or psychiatry (treatment) settings	Cluster A e.g. a nursing home or hospice for people with addiction	Cluster B e.g. an addiction and/or mental health institute
Settings with a generic approach to SUD or psychiatry (not-specialized)	Cluster C e.g. a palliative consult team	Cluster D e.g. a general practitioner service or hospital

All potential participants will receive an information brochure, either directly (strategy 1) or after showing interest (strategies 2 and 3). This brochure includes two questions asking to self-report competencies in addiction and palliative care. The HCPs+ will also be asked to provide some demographic details, so that the researchers can assess whether enough diversity in the focus groups has been reached. Demographic data will also provide context for the interpretation of future results.

### Data collection

#### Patients and proxies

AE will conduct semi**-**structured interviews with patients and proxies, either at the place where the participant is currently living, e.g.in a healthcare institution. An interview is both a form of dialogue and bond between researcher and participant. This qualitative method offers room for the researcher to get to know the lives, worlds and experiences of the participants. Participants in return can fully express and explicate themselves as the interview guide we developed, will not be strictly structured or standardized [62, 64]. Participants will be encouraged to speak freely about emotions, thoughts, actions and experiences with living with incurable disease, a SUD and the palliative care they receive. The project group members in consultation with an ‘expert**-**by**-**experience’ and a patient decided it was best to draw up an interview guide using direct and simple terminology and questions, especially in view of the patients. In addition, the researcher will ask the central HCP what end-of-life care terminology is best suited for an individual patient, so as to increase recognition during the interview.

Interviews with these vulnerable patients will be limited to 1 h, in line with the way patients often talk about their lives: briefly and to the point. The proxy interviews will be limited to one**-**and**-**a**-**half hour. However, interviews can be extended or, if necessary, continued at a later date by mutual agreement between the participant and the interviewer. Patient and proxy are both allowed to bring somebody to the interview, if this makes them feel more comfortable.

If possible, the researcher will request the patient’s demographics from the central HCP before the scheduled date of the interview. This will allow reducing the duration, and thus the burden of the interview and will provide context to the results. Proxies will be asked to provide a few demographics at the start of the interview. Before starting of the interview, the interviewer will briefly explain the rationale for this research as well as the purpose of the interview. Only the interviewer’s name, occupation and professional contact details will be disclosed. Both patient and proxy will be, again, asked to recount the patient’s illness trajectory. This will serve to check whether they are aware that the patient is in the palliative care phase. If this does not appear to be the case, the interviewer needs to be flexible and adapt the interview to the patient’s current health situation.

During an interview, the interviewer will write down keywords on cards so as to be able to provide feedback on the interviewee’s information provision. If necessary, the interviewer will also write field notes to be able to probe more efficiently. Furthermore, the interviewer will bring so-called association cards that contain pictures which can be used as metaphors when a participant is struggling to find the right words for experiences [65]. These will be used only as a back-up method for data collection.

The main- and subtopics addressed in the interview guide for patients and proxies are presented in Table 2. These are the areas around which the open-ended questions were developed. Subtopics are provided because the project group members know from experience that this patient group, in general, is not very talkative.

**Table 2:** Main and sub-topics of patient and proxy* interviews

Main topics	Subtopics
1. Healthcare	Care network, organization of care, attention for different dimensions, HCPs’ coping with a SUD, communication with HCPs
2. Physical health	Pain (control), symptom (burden), impact of a SUD
3. Social aspects	Social network / isolation, communication with others, impact of a SUD
4. Psychological and existential issues	Life values, sources of strength, future, place of death, impact of a SUD
5. Proxy experiences	Contact between HCPs and proxy, care for proxy, involvement in planning and decision**-**making and, needs in life and care.

^*^Proxies will be asked questions from domains 1 to 4 relating to the patient’s situation

#### Healthcare professionals, ‘experts-by-experience’ and volunteers

Focus group interviews will be led by MG and AE at centrally situated venues in the Netherlands. This method is suitable for grasping a diversity of opinions and experiences [66] and collecting a large amount of data in a short time. The group interviews will take approximately 2 h. A semi**-**structured interview guide will be used [62, 64]. Main topics of the guide are: 1) Content of care on the palliative care dimensions (physical, psychosocial and spiritual, and pain) and existential issues; 2) Organization of palliative care for patients with SUD+; 3) Communication with patients; 4) Care for proxies and; 5) Knowledge and competence, relating to palliative care and SUD+. These are the areas around which the researchers developed open-ended questions. Subtopics are not provided. Issues that are important to HCPs+ are expected to emerge from the discussions and the open-ended and follow**-**up questions. Each main theme, however, was provided with contributive examples, serving as basis for potential probing questions in case nothing came up during the interviews, e.g. cooperation or identification of palliative care phase for the theme ‘organization of palliative care for patients with SUD+’.

The focus group sessions will be concluded with what is known as a strengths, weaknesses, opportunities and threats (SWOT) analysis. This analysis is suitable for delivering practical and clear results. Strengths and weaknesses refer to the internal environment of HCPs+’ workplace. These are either positive or negative attributes that stimulate or hinder the development of palliative care for patients with a SUD, e.g. personnel or location. Opportunities and threats are positive or negative conditions outside the workplace that stimulate or hinder the development of palliative care for patients with a SUD, e.g. stigmatization or political environment [67–69]. The workings of the SWOT analysis will be explained, after which the HCPs+ will be invited to state what SWOT-elements of current palliative care for patients with a SUD+ they find most important with regard to the five main interview topics. Participants will be given sticky notes on which they can write down these elements and which they can stick to five posters. We purposively have chosen for a combination of both a group interview and a concluding SWOT **analysis**, as the latter will serve as a summary of what has been discussed and a prioritization of the most important findings.

### Data analysis

#### Patients and proxies

Researchers (AE and MG) will use a two**-**stepped thematic content analysis, consisting of a directed and a conventional approach [70]. In the first step, coding is quite directed and deductive: main themes or main codes **-** derived from the research questions **-** will be linked to the relevant parts of the first interview transcripts, until inter coder reliability is reached [71]. This is an initial step in organizing the data. These main themes are: needs in personal lives, healthcare needs, healthcare problems, positive experiences within care and healthcare improvements. The second step consists of applying so**-**called ‘baby–codes’ within the main themes of the first interviews. These codes will be applied to words or text segments that capture concepts or thoughts relevant to the research questions. By staying as close as possible to the original data, we will create inductive codes that contain experiences from the participants’ perspectives. This is a way of avoiding over interpretation by the researcher. Afterwards, these ‘baby**-**codes’ are clustered into categories or subthemes, based on how the ‘baby**-**codes’ are related with each other and a particular main theme (axial coding). Each subtheme then is provided with a description. In this way, a codebook is created which will serve as a coding strategy for the analysis of the remaining interviews. The analysis process will be iterative: while analyzing, codes and relations between them can be continuously and repeatedly updated or adapted with new data (collected by the interviews) and critical and reflective questions that arise while analyzing [70, 72, 73].

After data analysis, the main researcher will present the findings to a similar patient group: residents of a Salvation Army nursing home specialized in complex care and treatment. These residents are in a better health state, but will meet many of the inclusion criteria applied to the study group. As a surrogate “member check”, they will be asked to what extent they recognize themselves in the results of the whole study group. The purpose of this surrogate “member check” is thus to more or less validate the study results [74]. The project group choose this surrogate “member check” instead of asking the participating patients from the original interviews, for a member check, because from practical and clinical experience it appeared it would be too burdensome to ask patients twice. Also, participating twice could withhold patients from participation at all.

#### Healthcare professionals, ‘experts-by-experience’ and volunteers

The interview transcripts of the focus groups will be analyzed in a similar way as the patient and proxy interviews. The main and subthemes, however, will be different and constitute the fives topics used in the focus group interviews. Again, a codebook will be created [70]. Afterwards, a matrix will be constructed per main theme, including the SWOT-elements mentioned.

Regarding the patient, proxy and focus group interviews, two researchers (AE and MG) will analyze the data until consensus about the codebooks can be established. Then, AE alone will continue the coding process. In this way interpretation and researcher bias is minimized and validity is strived for. Still, AE and MG will frequently join each other for discussions relevant to the analysis process. Data analysis is computer**-**aided by the qualitative research software Atlas.ti version 7.

### Ethical considerations

Eligible participants will receive an information brochure containing the study’s goal, content, details of participation and mentioning that they can withdraw from the study at any time. They will be given the opportunity to put questions to the researcher and are given a minimum of a week to decide about participation. In case they have not responded within this week, AE will contact them. Before the interview starts, informed consent will be signed by the participant and researcher. The participant thereby states to: 1) agree that the interview will be audio**-**recorded; 2) understand the information provided; and 3) participate voluntarily. Participants, will receive a small gift at the end of the interview.

Participants’ names and demographics and interview data will be stored in encrypted databases. Participants’ names and other details will be assigned a code in a computer file, which is protected by a password only known by the researchers. In future publications, interview transcripts and the “member check”, only these codes will be used to guarantee participants’ anonymity and privacy. Patients and proxies will be invited to receive a transcript of their interview.

The risk of taking part in this study was categorized as ‘negligible’ [75]. Still, several steps have been undertaken to minimize the burden for the patients especially. First, an ‘expert**-**by**-**experience’ and patient with a SUD+ have helped drawing up the study protocol, the interview guides, recruitment and inclusion strategy, and information brochures. Their ideas about burden, structure, themes, terminology and understandability of the interview questions and information brochure were taken seriously. Second, interviews will be restricted in duration and will be held only once. Furthermore, the interviewer will ask the patients and proxies repeatedly whether they are still willing and able to continue. Third, participants can end participation at any time, without being obliged to give a reason. Fourth, the researchers are trained and experienced in communicating with vulnerable patient groups. Additionally, if the interviewer notices that a patient or proxy is struggling emotionally, she will tell so and discuss with the participant whether or not to continue the interview. Participants who are sad, anxious or show other negative emotions after the interview will be recommended to contact their own social or medical network or to find support from the person, if any, they brought to the interview. If there is no such network, the interviewer could potentially intervene by proposing to call the participant’s general practitioner. Lastly, the surrogate “member check” in which the researcher will present her findings during a monthly meeting for residents of the Salvation Army care home. These residents join voluntarily.

Although speculating about long-term consequences might be challenging, we believe that the psychological risks of participating in this study are minimal. Participation might even be experienced as beneficial and positive. Patients in a palliative care phase may find it therapeutic to share their problems or to contribute to research and thereby support other patients. Also, studies have shown that patients and their proxies find talking about death and dying (timely) helpful in getting a better grip on the situation [76, 77] and could bring rest, connection, an actualization of wishes and less anxiety [78]. For healthcare professionals, volunteers and ‘experts**-**by**-**experience’, study participation can be beneficial because they contribute to the advancement of palliative care for a vulnerable patient group. Also, they will reflect on and be inspired by a comparison of their own experiences, practices and collaboration and those of other HCPs+.

## Discussion

### Strengths

The network of the project group is diverse and will be of great help in recruiting patients as well as HCPs+ from Clusters A, B and C (see Table 1). Furthermore, this project allows to combine empirical results from different points of views of people involved with palliative care for patients with a substance use disorder and multiple problems. Finally, the study will grasp the multidimensional aspects of healthcare and care experiences.

### Challenges

Recruitment of patients probably will be hard because they often do not show up for appointments and might not be tempted to talk (extensively) about issues that might trigger emotions, e.g. shame [39, 41–43, 79]. It is likely that HCPs+ from Cluster D will be harder to recruit, as patients with SUD are not always visible and experiences with this patient group might be rare. Also, recruiting proxies might be a challenge as the social networks of patients are often limited.

## Conclusion

This study protocol article provides insights into ways to conduct research with vulnerable research participants, by offering a detailed description of the study design and the methodological approaches. The outcomes will include an explicit recognition of problems, needs, good examples and improvements experienced or suggested by patients with a SUD+, proxies, healthcare professionals, volunteers and ‘experts**-**by**-**experience’. The study will provide valuable knowledge on how to improve clinical practice, and patient and proxy care. Additionally, the findings will lie at the foundation for an education program for Dutch HCPs who are dealing with patients with SUD+ in a palliative care phase. In the end, this project aims to achieve an optimal end-of-life phase and a dignified dying process for patients with a substance use disorder and multiple problems.

## Additional file

Additional file 1: Case Study SUD+. (DOC 27 kb)

## Abbreviations

COREQ: COnsolidated criteria for REporting Qualitative research
DSM**-**V: Diagnostic and Statistical Manual of Mental Disorders, five
ESBL: Extended**-**Spectrum Beta**-**Lacatmases
GHB: Gamma**-**Hydroxybutyric acid
HAART: Highly Active Anti-Retroviral Therapy
HCPs: healthcare professionals
HCPs+: healthcare professionals, volunteers and ‘experts-by-experience’
SUD+: Substance Use Disorder and multiple problems
SUD: Substance Use Disorder
SWOT: Strengths, Weaknesses, Opportunities, Threats
