# Supplementary material for: Palliative care for patients with a substance use disorder and multiple problems: a study protocol
Source: BMC Palliat Care. 2018 Aug 3;17:97. doi: 10.1186/s12904-018-0351-z (PMC6091086; doi:10.1186/s12904-018-0351-z)
Supplement: Supplementary file 1 — Case Study SUD+. (DOC 27 kb) [file 12904_2018_351_MOESM1_ESM.doc]

**Additional file 1** Case study

**CASE STUDY**

Charlotte (pseudonym) is a 52**-**year**-**old woman. Since 2014 she is living in a specialized nursing home for people with complex care needs and treatment. Due to her background in addiction (a recent history of cocaine and heroin) and homelessness, it has been hard to find her a suitable care place in regular care. Charlotte is ‘doubly’ diagnosed with psychiatric problems, substance abuse and severe somatic problems (HIV**-**infection, cervix carcinoma, hepatitis C, sensorimotor polyneuropathy, amputated foot, decubitus, Extended Spectrum Beta**-**Lactamase (ESBL)**-**positive and minor kidney**-**problems). Non**-**healing decubitus wounds are her biggest problem. Charlotte uses several medicines, including methadone. She has an extensive medical history and previous hospital admission showed that she is resistant against multiple antibiotics.
 Charlotte is wheelchair**-**dependent and needs intensive support with activities of daily living. Despite, she functions quiet well: she always appears neat and communication with the healthcare staff is friendly. She has a strong liking for autonomy. Recently, she had two episodes of severe infection and delirium and is advised to stay in bed to stimulate wound healing. However, Charlotte is not complying. She wants to keep in touch with her social environment and to go out for a smoke: these things, she says, make up for her quality of life.

Antibiotic wound treatment becomes harder due to the multiple resistances. The decubitus takes a turn for the worse and Charlotte increasingly becomes bedridden. Proactive care planning is discussed with Charlotte and her sister. Charlotte clearly takes charge: she refuses a DNR**-**order and prefers to be treated at home (the nursing home) instead of being admitted to a hospital. Talking about future deterioration or dying, though, is out of the question. She says to have faith in the future. Even when waking up anxious or when the spiritual caregiver reads her poems and Bible texts, she does not open up about end**-**of**-**life issues.

Meanwhile, pain medication has to be adapted and fentanyl patches are started, which after six weeks reach 300mcg. Also, escape-medication (oxycodon) is provided, which is later replaced with oral morphine as swallowing becomes a struggle. Providing appropriate pain treatment remains problematic until her death.
 About three weeks before her death, Charlotte opens up and agrees to retract the DNR**-**order. Together with the health professionals it is decided she will not be sent to the hospital and will be taken care of in a loving way. Although weak, Charlotte maintains her strength: a few weeks before she passes away, she still has her hair done and smokes cigarettes outside, in the sun. She does not want to stop Highly Active Anti-Retroviral Therapy (HAART), because “then”, she says, “I will get Aids”. Charlotte is able to properly say final goodbyes to her mother, sister, ex**-**husband and daughter. This gives her peace.
 In September 2016, Charlotte’s Palliative Performance Scale drops to 20%. From that moment, the healthcare professionals start with sedation. She appears anxious and confused. With her agreement, the sedation is intensified. Several changes in pain medication have to be made to assure that Charlotte is at peace and as pain**-**free as possible. On the 20^th^ of September 2016, Charlotte dies peacefully.
